# Factors Influencing Acceptance of Post-Mortem Examination of Children at a Tertiary Care Hospital in Nairobi, Kenya

**DOI:** 10.5334/aogh.2504

**Published:** 2019-07-03

**Authors:** Milka Bunei, Peter Muturi, Fred Otiato, Henry N. Njuguna, Gideon O. Emukule, Nancy A. Otieno, Jeanette Dawa, Sandra S. Chaves

**Affiliations:** 1 Influenza Program, IHRC, Nairobi, KE; 2 Kenya Medical Research Institute (KEMRI), Center for Global Health Research, Kisumu, KE; 3 Influenza program, Division of Global Health Protection, Centers for Disease Control and Prevention, Nairobi, KE; 4 Washington State University, Global Health Program, Nairobi, KE; 5 Influenza Division, National Center for Immunization and Respiratory Diseases, US Centers for Disease Control and Prevention, Atlanta, GA, US

## Abstract

**Background:**

Clinical autopsies are not often part of routine care, despite their role in clarifying cause of death. In fact, autopsy rates across the world have declined and are especially low in sub-Saharan Africa.

**Objectives:**

We set out to identify factors associated with acceptance of pediatric autopsies among parents of deceased children less than five years old, and examined local preferences for minimally invasive tissue sampling (MITS) procedures during post-mortem (PM) examinations.

**Methods:**

From December 2016 to September 2017, we contacted 113 parents/next of kin who had been previously approached to consent to a PM examination of their deceased child as part of a Kenyan study on cause of death. Interviews occurred up to three years after the death of their child.

**Findings:**

Seventy-three percent (83/113) of eligible study participants were enrolled, of whom 62/83 (75%) had previously consented to PM examination of their child. Those who previously consented to PM had higher levels of education, were more likely employed, and had more knowledge about certain aspects of autopsies than non-consenters. The majority (97%) of PM consenters did so because they wanted to know the cause of death of their child, and up to a third believed autopsy studies helped advance medical knowledge. Reasons for non-consent to PM examination included: parents felt there was no need for further examination (29%) or they were satisfied with the clinical diagnosis (24%). Overall, only 40% of study participants would have preferred MITS procedures to conventional autopsy. However, 81% of autopsy non-consenters would have accepted PM examination if it only involved MITS techniques.

**Conclusion:**

There is potential to increase autopsy rates by strengthening reasons for acceptance and addressing modifiable reasons for refusals. Although MITS procedures have the potential to improve autopsy acceptance rates, they were not significantly preferred over conventional autopsies in our study population.

## Background

Notwithstanding recent improvements in diagnostic technologies, full-body autopsies remain the gold standard in determining cause of death. This is because discordancy rates between clinical and autopsy diagnoses for communicable and non-communicable conditions range from 17% to 65%, and up to 40% of post-mortem (PM) examinations reveal significant information regarding cause of death that was not previously known [1,2,3,4].

Clinical autopsies are not frequently performed as part of routine care [4,5]. Furthermore, autopsy rates across the world have declined, and rates are especially low in sub-Saharan Africa, where low perceived benefit, fears of body disfigurement, the practicalities of funeral arrangements, and cultural beliefs lead to autopsy refusals by next of kin [4,5,6,7]. Aside from refusals to consent by next of kin, the cost and expertise of carrying out a full clinical autopsy is an impediment to its use, especially in Africa [8,9]. There are few trained pathologists in the African region, leading to insufficient human resource capacity to cover autopsy workloads [8]. In these settings, minimally invasive autopsies (MIA), which involve less invasive PM examination procedures such as minimally invasive tissue sampling (MITS) techniques, have been shown to provide a cause of death determination in four out of five cases [10]. However, they are more sensitive in accurately determining cause of death in infectious conditions as compared to non-infectious conditions [10,11]. MIAs do not require removal of organs or major incision marks on the body. As it is a less intensive procedure, the turnaround time for results can be faster and it does not lead to major delays in burial, which would be particularly important in some communities where these constitute reasons for refusal [12,13]. In Kenya, PM examinations are not frequently performed [14], although with adequate community mobilization, autopsy acceptance rates of 80% have been observed [15].

We set out to identify local factors associated with consent and refusal of pediatric autopsies at a tertiary teaching and referral hospital in Nairobi, Kenya, and examined local preferences for minimally invasive tissue sampling (MITS) techniques over the conventional autopsy. Understanding the reasons for consent and refusal, as well as preferences for MITS, could assist health authorities in developing strategies to increase local autopsy rates in order to obtain more accurate data on the causes of mortality and thereby guide health policies.

## Methods

### Study setting and design

The study was done among bereaved parents and family members (hereafter referred to as next of kin) who had sought care for their sick child at Kenyatta National Hospital (KNH), Kenya’s largest public teaching and referral hospital, located in the capital city, Nairobi. Eligible participants were bereaved next of kin who had previously been approached to be part of the Pediatric Respiratory Etiology Surveillance Study (PRESS) carried out from August 2014 to December 2015. PRESS was designed to determine the cause of death among deceased children under five years of age who had been hospitalized with a respiratory illness [16]. As a component of PRESS, grief counseling was offered to bereaved next of kin before they were approached to consider PM examination in the form of both conventional autopsy and MITS procedures. Grief counseling consisted of one or more therapy sessions between family members and a trained grief counselor/psychologist.

We carried out a retrospective cross-sectional survey, from December 2016 to September 2017, to collect information from next of kin who had received counseling as part of PRESS, regardless of whether they consented to PM examination of their child or not. The interview questionnaire was semi-structured with both open- and closed-ended questions. These interviews with family members took place up to three years after the deaths had occurred.

### Study procedures

We invited next of kin to participate in face-to-face or phone interviews where the questionnaire was administered in either English or Swahili after obtaining consent. Up to three attempts to contact the next of kin by phone were made during weekdays and at least one call was made over the weekend. The interviewer posed the question to the study participant and filled in their responses directly into an electronic password-protected database created using Epi Info, version 7.

### Data management and analysis

Study participants received unique identification numbers and we entered de-identified data into the electronic password-protected database. We used descriptive analysis to summarize the characteristics of study participants. We grouped responses from open-ended questions into similar themes and presented responses in the form of counts. Chi-square or Fisher exact tests were used when comparing proportions and to assess factors associated with PM acceptance or refusal.

### Ethical considerations

Ethical approval to conduct the study was obtained from the Kenya Medical Research Institute (KEMRI) Scientific and Ethics Review Unit (reference number 3265). The Institutional Review Board of the Centers for Disease and Control Prevention (CDC) relied on KEMRI’s review. Approval was given to obtain verbal consent during interviews.

## Results

### Description of survey respondents

For this study, we attempted to contact all 113 next of kin who received bereavement counseling after the death of their child as part of PRESS. These included 66 persons who had previously agreed to PM examination of their child and 47 who did not. Eighty-three (73%) out of the 113 targeted individuals were interviewed. Of the 30 individuals who did not participate in the study, eight had been contacted but declined to participate, while 22 could not be reached. Of those who were interviewed, there were 51 face-to-face interviews and 32 phone interviews. Of the 83 study participants, 75% (62/83) had previously consented to PM examination of their child (Table 1). The median age of study participants was 32 years (range: 22 to 63 years), and 61% were male. The majority (92%) of participants were parents while the remainder were part of the deceased child’s extended family. Most (67%) respondents were married, 27% were single and 6% separated. The majority (98%) of those interviewed were Christians. One participant was Muslim and the remaining participant was not affiliated with any religion. All respondents had completed primary school education, and 84% were employed. In the decision-making process about whether to conduct a PM examination, fathers were most commonly consulted (73%), followed by mothers (36%) and then extended family members (19%). Of note, none of the respondents who participated in the study had been asked to consider PM examinations by the doctor who treated their deceased child (Table 1).

**Table 1:** Characteristics of family members participating in a survey investigating factors associated with acceptance of post-mortem examination, Nairobi, Kenya (n = 83).

Characteristics of respondents				Previously consented to post-mortem examination of deceased child				
			
		Total (n = 83)		Yes (n = 62)		No (n = 21)		

		*n*	*%*	*n*	*%*	*n*	*%*	p value*

1	Age category							
	*20–30*	27	32.5%	22	35.5%	5	23.8%	
	*31–40*	42	50.6%	32	51.6%	10	47.6%	
	*Over 40*	14	16.9%	8	12.9%	6	28.6%	0.22
2	Relationship to the deceased child							
	*Mother*	27	32.5%	21	33.9%	6	28.6%	
	*Father*	49	59.0%	36	58.1%	13	61.9%	
	*Extended family*	7	8.4%	5	8.1%	2	9.5%	0.93
3	Sex of parent/guardian							
	*Male*	51	61.4%	38	61.3%	13	61.9%	
	*Female*	32	38.6%	24	38.7%	8	38.1%	0.96
4	Marital status of parent/guardian							
	*Single*	22	26.5%	16	25.8%	6	28.6%	
	*Married*	56	67.5%	43	69.4%	13	61.9%	
	*Separated*	5	6.0%	3	4.8%	2	9.5%	0.64
5	Religious affiliation of parent/guardian							
	*No religion*	1	1.2%	1	1.6%	0	0.0%	
	*Christian*	81	97.6%	60	96.8%	21	100.0%	
	*Muslim*	1	1.2%	1	1.6%	0	0.0%	N/A
6	Highest level of education							
	*Primary*	32	38.6%	23	37.1%	9	42.9%	
	*Secondary*	33	39.8%	21	33.9%	12	57.1%	
	*College/University*	18	21.7%	18	29.0%	0	0.0%	0.01
7	Employment status							
	*Employed – office work*	12	14.5%	12	19.4%	0	0.0%	
	*Employed – manual work*	20	24.1%	14	22.6%	6	28.6%	
	*Self-employed*	23	27.7%	18	29.0%	5	23.8%	
	*Casual*	15	18.1%	11	17.7%	4	19.0%	
	*Unemployed*	7	8.4%	2	3.2%	5	23.8%	
	*Homemaker*	5	6.0%	5	8.1%	0	0.0%	
	*Others*	1	1.2%	0	0.0%	1	4.8%	0.01
8	Were advised about the need for post-mortem by the doctor treating the child	0		0		0		N/A
9	Persons involved in making the decision regarding post-mortem examination of the deceased child^#^							
	*Father*	61	73.5%	47	75.8%	14	66.7%	0.50
	*Mother*	30	36.1%	26	41.9%	4	19.1%	0.38
	*Grandmother*	6	7.2%	1	1.6%	5	23.8%	
	*Grandfather*	7	8.4%	4	6.5%	3	14.3%	0.73
	*Aunt*	2	2.4%	2	3.2%	0	0.0%	
	*Uncle*	1	1.2%	1	1.6%	0	0.0%	
	*Other*	8	9.6%	6	9.7%	2	9.5%	0.9947

* Chi square test, Fischer exact test and test of proportion as appropriate; #Multiple responses permitted.

Next of kin who had agreed to PM examination of their child during PRESS (i.e., above-mentioned pediatric respiratory mortality study) were more likely to have tertiary education, be employed in an office or be home makers, while PM non-consenters were more likely to be unemployed (p-value = 0.01) (Table 1).

Participants were knowledgeable on most aspects of PM examinations; however, more than half of the respondents (61%) did not know that autopsies also included external examination of the body. The majority of participants (88%) felt that relatives should have the right to request PM examinations and that doctors should encourage relatives to request PM examinations (82%). Less than a fifth of all study participants believed that PM examinations were disrespectful to the dead (Table 2).

**Table 2:** Proportion of family members who answered “yes” in a survey investigating factors associated with post-mortem examination of their deceased children, Nairobi, Kenya (n = 83).

Item		Number and proportion of respondents who answered yes to survey questions						

		Total survey respondents (n = 83)		Survey respondents who previously consented to post-mortem examination (n = 62)		Survey respondents who previously did not consent to post-mortem examination (n = 21)		

		n	%	n	%	n	%	p value*

	*Knowledge of post-mortem examination*							
1	Do you think post-mortem examination can help identify the actual cause of death?	75	90.6%	59	95.2%	16	76.2%	0.02
2	Do you think post-mortem examination involves external examination of the dead body?	32	38.6%	23	37.1%	9	42.9%	0.76
3	Do you think post-mortem examination involves internal examination of the dead body?	76	91.6%	57	91.9%	19	90.5%	0.84
4	If death occurs before the patient arrives in a hospital, for example person died at home, do you think it is important to perform a post-mortem examination?	70	84.3%	56	90.3%	14	66.7%	0.02
	*Patient’s rights regarding post-mortem examination*							
5	Do you think relatives should request doctors to perform post-mortem examination?	73	88.0%	54	87.1%	19	90.5%	0.70
6	Do you think doctors should encourage relatives to request post-mortem examination?	68	81.9%	53	85.5%	15	71.4%	0.21
	*Attitudes and preferences regarding post-mortem examination*							
7	Do you think performing a post-mortem examination is disrespectful to the deceased?	14	16.9%	6	9.7%	8	38.1%	0.23
8	Do you think post-mortem examination should be part of the end-of-care services given to every person dying at the hospital?	30	36.1%	26	41.9%	4	19.1%	0.38
9	Would you advise other parents (people) to accept/request for post-mortem examination in case their loved ones die?	67	80.7%	62	100.0%	5	23.8%	<0.01
10	There are minimally invasive techniques to obtain post-mortem specimens for laboratory investigations such as using needles to collect tissue samples. Though not very comprehensive, these techniques may be used instead of fully opening up the body to collect specimens. Would you prefer these minimally invasive techniques?	33	39.8%	16	25.8%	17	81.0%	<0.01

* Test of proportion.

For the most part, the two groups did not differ substantially in regards to knowledge, attitude and preferences related to PM examinations. As compared to those who previously declined PM examination of their child, PRESS PM consenters were more likely to think that the procedure could assist in identifying cause of death (95% vs. 76%, p-value = 0.02), and that it could be important in cases of death in the community or on arrival at a hospital (90% vs. 67%, p-value = 0.02). Substantial differences between the two groups were observed in recommending PM examinations, where only 24% of those who previously declined PM examination would advise parents of deceased children to undertake the procedure as compared to 100% of those who previously accepted PM examination (p-value <0.01). Overall, only 40% of study participants would prefer MITS techniques for PM examination. However, 81% of those who previously did not consent to PM examination would have consented if the procedure had only involved MITS procedures (p value <0.01) (Table 2).

When we explored reasons for accepting PM examinations during PRESS, nearly all (97%) said they wanted to know the cause of death of their child. Up to a third of respondents participated in the study to help advance medical knowledge. One mother declared that she did not want to see another mother go through the pain she had experienced. Another parent commented that if it would help others, so be it, as they did not lose anything from participating in the study. For other respondents, reasons for PM examination included legal requirements due to the circumstances surrounding death or concerns for family health problems – the family wanted to know whether the cause of death was associated with genetic problems or wanted to prevent a similar illness in their future children (Table 3). Some of the next of kin who consented to the autopsy during PRESS did so despite believing it was disrespectful to the dead (n = 6; Table 2). Among these individuals, the reasons for consenting included: to find out the actual cause of death (n = 6), to advance medical knowledge (n=2), and the services were free (n = 1).

**Table 3:** Reasons that family members accepted post-mortem examination of their deceased children in Nairobi, Kenya (n = 62).

Reason	Number	Percentage

To know actual cause of death	60	96.8%
Information from the post-mortem examination will help advance knowledge	18	29.0%
To know if the cause of death was genetic	3	4.8%
It was a legal requirement	2	3.2%
To help prevent similar illness in deceased child’s siblings	2	3.2%
To understand more about a genetic condition that the child had	1	1.6%
To clear oneself of suspicion of contributing to the child’s death	1	1.6%
Needed a detailed written report of the post-mortem examination findings	1	1.6%
Post-mortem examination services were free	1	1.6%
Suspicion of negligence from medical staff	1	1.6%

*Note:* Multiple responses permitted.

Among those who had refused to participate in PRESS, 29% chose not to consent to the autopsy because they felt there was no need for further examination after the death of their child, and 24% were satisfied with the clinical diagnosis. Religious and cultural beliefs (19%), fear of organ removal (14%) and disappointment with the care the child had received while hospitalized (14%) also contributed to refusals (Table 4).

**Table 4:** Reasons that bereaved family members declined post-mortem examination of their deceased children in Nairobi, Kenya (n = 21).

Reason	Number	Percentage

The child had died; therefore there was no need for a post-mortem examination	6	28.6%
Was satisfied with the clinical diagnosis	5	23.8%
Post-mortem examination is forbidden in their culture or by their religion	4	19.0%
Was afraid body parts would be removed	3	14.3%
Was angry and disappointed by the care the child received at the hospital	3	14.3%
Was concerned it would delay funeral arrangements	2	9.5%
Chose to accept God’s will	2	9.5%
Resistance from family members	2	9.5%
Child was too young for a post-mortem examination	1	4.8%
Too painful to talk about at the time	1	4.8%
Don’t remember the reason	1	4.8%

*Note:* Multiple responses permitted.

## Discussion

In this study, we interviewed family members who had received bereavement counseling following the death of their child and were asked for permission to conduct a PM examination to identify their child’s cause of death. We included those who consented to the PRESS study and those who did not. Regardless of their participation in the previous PM study, a majority of family members in both groups thought that PM examination could help identify the cause of death. Despite the small sample size, 81% of parents who previously refused PM examination of their child would most likely have accepted it if MITS was used instead of conventional autopsy. MITS could be an alternative approach to PM examination to determine infectious causes of death when cultural driven factors regarding timing of burial or cutting of the body preclude the use of conventional autopsy.

In a social behavioral study conducted in five low- and middle-income countries in Asia and Africa, the hypothetical acceptance of MIA (which includes MITS techniques) on deceased relatives was >70% [14]. In Bangladesh, a predominantly Muslim country, MITS techniques were deemed more acceptable than conventional autopsies because they did not require major delays in burial, removal of organs, or cutting or stitching of the body [12]. In a Belgian study, researchers were also able to demonstrate that Muslim parents preferred MITS to the conventional autopsy [17]. In our study setting, where the population was mainly Christian and the main reasons for decline did not include concerns of opening up the body, MITS techniques would likely not have as much as an effect on improving overall PM examination acceptance rates (previous studies have described conventional autopsy acceptance rates as high as 80% in rural Kenya [15]). Nevertheless, acceptance of MITS is encouraging especially in resource-constrained set-ups like ours, where routine standard autopsy is not always available.

In Nigeria, younger parents are less likely to consent to PM examination of their children than older parents [7], while non-Muslims and those with higher levels of education are more likely to consent [9]. In Belgium, maternal age, parity and education has no association with autopsy acceptance rates [17]. In our study, we were able to demonstrate that unemployed people, those with less education and those with less knowledge about autopsies were least likely to consent to autopsies. This suggests that the effects of socio-demographic factors on autopsy acceptance rates are largely context specific, and generalizations from studies conducted in different settings should be avoided.

Similar to our findings, one of the most common reasons given by family members for consenting to autopsy studies is to know the cause of death [9,14]. Aside from finding out why their child had died, parents are also motivated to consent to autopsies to prevent similar illnesses in their other children and by a strong sense of altruism [18,19,20]. Parents hope to advance medical knowledge in order to help other families avoid the suffering associated with the death of a child. Clinicians could make use of these motivators to increase autopsy rates.

Studies have shown that PM examinations are often not conducted because the deceased’s family members are not asked to consider this option [21,22]. In our study, none of the participating families were advised to undertake a PM examination by their attending doctor. Simple measures such as involvement of the attending physician in discussions with relatives about PM examination have the potential to significantly increase autopsy rates, as documented previously [23].

The majority of those who declined PM examination of their child did so because they felt there was no need for it (they had already lost their child) or because they were satisfied with the clinical diagnosis received from the attending clinician. Similarly, in a study conducted in Scotland, parents felt that there was no need for PM examination given that their child had died [18]. In Zambia, most parents declined PM examination because they did not see the benefit of it [7]. This seems to be a common theme: pragmatism surrounding death. Nonetheless, PM studies are important to identify potentially preventable deaths and advise health policies and medical staff on improvements in patient management [4,24]. Health care providers’ perceptions of the difficulties in obtaining consent from relatives, the administrative challenges associated with autopsies, perceived poor quality of autopsies conducted, delays in obtaining autopsy reports, fears of revealing clinical mismanagement of patients and the threat of malpractice litigation are some of the reasons health care providers are hesitant to talk to family members about PM examinations [9,14]. Improving autopsy rates would therefore require interventions targeted at concerns raised by the clinician as well as the deceased’s family members.

Our study had some limitations. Although we attempted to contact all the families previously approached for the PRESS study, most of our respondents were families who accepted autopsy investigation of their deceased child. We had 94% of consenters and 45% of non-consenters from PRESS participating in this survey. Those not interviewed could have different perspectives about PM examination (e.g., not inclined to agree to PM examination even if MITS procedures are used) or different demographics. The responses elicited from the phone interviews versus face-to-face interviews could also have been different. Moreover, our findings need to be interpreted within the context of the study setting. The study was based at a tertiary care referral hospital; most of the families would have sought care in other health care facilities before arriving in the study hospital and may have different attitudes and expectations towards understanding the cause of death of their child when compared to the general population and cases of death in the community. Even so, the study offers an insight into this population and may guide future initiatives to investigate causes of death in similar settings.

## Conclusion

A desire to know the cause of death and an altruistic attitude among the deceased’s family members are great motivators in consenting for autopsy. There is potential to increase autopsy rates by strengthening reasons for acceptance and addressing modifiable reasons for refusals. Although MITS techniques have the potential to improve autopsy rates, they may not be significantly preferred over conventional autopsy in settings where opening up the body and removal of organs are not one of the major reasons for refusal by the deceased’s next of kin.

## Disclaimer

The findings and conclusions in this report are those of the authors and do not necessarily represent the official position of the Centers for Disease Control and Prevention.
